# The Innovative Health Initiative public–private partnership: a catalyst for big data-driven health research and innovation

**DOI:** 10.3389/fmed.2025.1554948

**Published:** 2025-04-01

**Authors:** Elisabetta Vaudano

**Affiliations:** Innovative Health Initiative Joint Undertaking, Brussels, Belgium

**Keywords:** collaboration, data access, quality, patients, trust, big data, public–private partnership, health

## Abstract

The use of “Big Data” in health research could be a game changer to revolutionize the field and drive innovation. However, the complexity of health data and of the healthcare ecosystem makes big data applications challenging. To overcome these challenges, it is essential that all stakeholders, both data owners and users, feel comfortable with the data use and the results of the analyses. This can only be achieved using a collaborative approach that prioritizes transparency and trust. The European Innovative Health Initiative (IHI) is a prime example of a successful public–private partnership that has achieved this. By fostering a culture of collaboration and trust, the IHI has enabled unprecedented access to large amounts of health data, improved data quality and standards, and created valuable resources and knowledge for progress in areas of unmet public health need. This perspective explores the IHI model and highlights examples from its projects that demonstrate its power as a catalyst for big data-driven health research and innovation. By sharing lessons learned and best practices, we aim to contribute to the development of a robust data-driven evidence base for the translation of research into improved patient care practices and products.

## 1 Introduction

The use of “Big Data” in health research has revolutionized the way we approach medical research, diagnosis, and treatment. Big data are extremely large and diverse collections of structured and unstructured data from sources such as electronic health records (EHRs), omics, imaging, and wearables. The progress in advanced analytics and artificial intelligence (AI) (most recently generative AI) has enabled using such rich sources of information to uncover hidden patterns, trends, and correlations within large datasets for a deeper understanding of complex phenomena and unprecedented speeding up of innovation (1).

However, health data are complex and sensitive, and the ecosystem of data owners and users is a mix of public and private stakeholders, making big data applications challenging. For success, it is necessary all actors feel comfortable with the data use and the results of the analyses. This can only be achieved using a collaborative approach based on transparency and trust among all stakeholders. Trust is the cornerstone of public–private partnerships (PPPs), such as those created by the European Innovative Health Initiative (IHI) (2), making these ideal instruments for addressing the challenges of using big data in health research, fostering collaboration, and driving innovation in this critical field. This perspective presents some examples from selected projects (Table 1) of what can be achieved through public–private collaboration.

**Table 1:** List of IHI projects cited in the text with the acronym, title, link to website (or, in the absence of this, to the factsheet on the IHI website), and a short description of project scope.

Acronym, Title, Website	Project scope
**AIMS-2-TRIALS** Autism Innovative Medicine Studies – 2 – Trials https://www.aims-2-trials.eu/	A large-scale European research project focused on autism spectrum disorders (ASDs). It aims to improve understanding, identify, and validate biomarkers, foster the development of new treatments, and create a clinical trial network for autism across Europe. The project seeks to advance personalized medicine approaches for individuals with ASDs, exploring autism’s development from prenatal stages to adulthood.
**BD4BO** Big Data for Better Outcomes https://bd4bo.eu/	A program aimed to improve the health outcomes of patients in Europe by maximizing the potential of big data through collaborative research projects. It created big data platforms focused on specific disease areas, including Alzheimer’s, blood cancers, cardiovascular diseases, and prostate cancer.
**BIGDATA@HEART** Big Data at Heart https://www.bigdata-heart.eu/	A project that developed a big data-driven platform for cardiovascular research, focusing on improving outcomes in heart diseases.
**BIGPICTURE** Central repository for digital pathology https://bigpicture.eu/	A project creating a European repository of 3 million digital pathology slides from both humans and laboratory animals, providing a comprehensive dataset for AI tool development across a broad spectrum of diseases and medical conditions where pathology is critical for diagnosis and research.
**CONCEPTION** Building an ecosystem for better monitoring and communicating of medication safety in pregnancy and breastfeeding: validated and regulatory endorsed workflows for fast, optimized evidence generation https://www.imi-conception.eu/	A project that created a pan-European network that systematically generates and disseminates reliable evidence-based information on medication safety during pregnancy and breastfeeding.
**DIRECT** Diabetes research on patient stratification https://www.ihi.europa.eu/projects-results/project-factsheets/direct	A project that identified biomarkers for the prediction of disease progression and treatment response to develop personalized medicine approaches for type 2 diabetes.
**EHDEN** European Health Data and Evidence Network https://www.ehden.eu/	A project aimed to harmonize European health data at a large scale (100 million) to a common model, facilitating large-scale analysis and research to improve patient care and outcomes. It created a unique large-scale federated European health data network for rapid, reproducible research.
**EMIF** European Medical Information Framework https://www.emif.eu/	A project that created a unified platform to discover, assess, and reuse healthcare data across Europe, focusing on Alzheimer’s disease and metabolic complications of obesity. It developed tools for data harmonization, ethical governance, and collaborative research.
**EPAD** European prevention of Alzheimer’s dementia consortium https://ep-ad.org/	A project aimed at facilitating the secondary prevention of Alzheimer’s disease (AD). It recruited over 2,000 participants for a longitudinal cohort study across Europe and developed a master protocol for AD secondary prevention-adapted trials.
**EPND** European Platform for Neurodegenerative Diseases https://epnd.org/	A project that is creating a sustainable European platform to accelerate biomarker discovery for neurodegenerative diseases by facilitating access to clinical samples and data across Europe.
**EQIPD** European Quality In Preclinical Data https://go-eqipd.org/	A project aimed at improving the quality and reproducibility of preclinical research data through standardized quality systems and educational resources. It developed guidelines, tools, and a certification process to enhance scientific rigor and data reliability.
**eTOX** Integrating bioinformatics and chemoinformatics approaches for the development of Expert systems allowing the *in silico* prediction of toxicities https://www.ihi.europa.eu/projects-results/project-factsheets/etox	A project that developed a comprehensive toxicology database and predictive models to improve drug safety assessment in the early stages of pharmaceutical development, reducing animal testing, and accelerating drug discovery. It built the basis for the eTRANSAFE project.
**eTRANSAFE** Enhacing TRANslational SAFEty Assessment through Integrative Knowledge Management https://www.ihi.europa.eu/projects-results/project-factsheets/etransafe	A project aimed to enhance translational safety analysis and prediction. It developed an integrated database and computational tools to improve drug safety assessment using preclinical and clinical data from pharmaceutical companies and public sources.
**EU-AIMS** European Autism Interventions - a Multicentre Study for Developing New Medications https://www.ihi.europa.eu/projects-results/project-factsheets/eu-aims	A project aimed at accelerating the development of new treatments for autism spectrum disorders (ASDs) through comprehensive research and collaboration. It created one of the world’s largest autism databases, containing extensive medical information on individuals with ASDs. It built the basis for the AIMS-2-TRIALS project.
**EUROPAIN** Understanding chronic pain and improving its treatment https://www.ihi.europa.eu/projects-results/project-factsheets/europain	A project aimed to improve understanding of pain mechanisms and accelerate analgesic development. It transformed the neuropathic pain field through collaborative research, enhancing preclinical and clinical studies via novel animal models and objective pain measures as well as translationally validated novel drug targets.
**FAIRPlus** FAIRPlus https://fairplus-project.eu/	A project aimed to improve data sharing in life sciences by developing tools and guidelines to make research data findable, accessible, interoperable, and reusable (FAIR). It established a robust methodology for prioritizing and selecting datasets for FAIRification, providing practical guidance to improve FAIRness for existing and new datasets.
**HARMONY ALLIANCE** Healthcare alliance for resourceful medicines offensive against neoplasms in hematology https://www.harmony-alliance.eu/	Alliance of the two projects HARMONY and HARMONY plus that created a big data analytics platform to accelerate the development of more effective treatments for blood cancer patients.
**HIPPOCRATES** Health initiatives in psoriasis and psoriatic arthritis consortium European states https://www.hippocrates-imi.eu/	A project aimed at improving early diagnosis, prevention, and personalized treatment of psoriatic arthritis (PsA) through advanced research on the molecular basis of PsA, data integration, and patient involvement.
**HYPORESOLVE** Hypoglycaemia - REdefining SOLutions for better liVEs https://hypo-resolve.eu/	A project aimed at redefining hypoglycemia in diabetes, investigating its causes, consequences, and economic impact. It developed a unique database, identified links between hypoglycemia and cardiovascular events, revealed inflammation’s role, and developed a quality-of-life measure for hypoglycemia in diabetes patients.
**IDERHA** Integration of heterogeneous data and evidence toward regulatory and HTA acceptance https://www.iderha.org/	A project aiming to create a scalable platform integrating diverse health data to improve patient outcomes, focusing initially on lung cancer. It seeks to enhance personalized care and support healthcare professionals, patients, and researchers.
**MELLODDY** Machine learning ledger orchestration for drug discovery https://www.ihi.europa.eu/projects-results/project-factsheets/melloddy	A project that developed a secure (blockchain-based) federated learning platform for drug discovery, enabling pharmaceutical companies to collaborate on machine learning models without sharing proprietary data. It successfully deployed the world’s first secure multi-task federated learning platform for drug discovery.
**NEWMEDS** Novel methods leading to new medications for depression and schizophrenia https://www.ihi.europa.eu/projects-results/project-factsheets/newmeds	A project focused on developing new methods for drug discovery in schizophrenia and depression. It created standardized animal models, imaging tools, and one of the largest databases of schizophrenia clinical trials, for improving drug development efficiency. It also made significant contributions to understanding genetic risk factors in schizophrenia:
**NEURONET** Efficiently networking European neurodegeneration research https://imi-neuronet.org/	A coordination and support initiative that developed different tools to enhance collaboration, synergy, and visibility across IMI’s neurodegenerative disorder research projects, aiming to maximize their collective impact.
**OPTIMA** Optimal treatment for patients with solid tumors in Europe through artificial intelligence https://www.optima-oncology.eu/	A project using AI to improve care for prostate, breast, and lung cancer patients. It aims to develop a GDPR-compliant, real-world oncology data platform, and AI-based decision support tools.
**PIONEER** Prostate cancer diagnosis and treatment enhancement through the power of big data in Europe https://prostate-pioneer.eu/	A project aimed to improve prostate cancer care by leveraging big data. It developed a comprehensive data platform to address key knowledge gaps in screening, diagnosis, and treatment and in integrating real-world datasets. It conducted analyses to enhance patient outcomes and healthcare efficiency.
**Screen4Care** Shortening the path to rare disease diagnosis by using newborn genetic screening and digital technologies https://screen4care.eu	A project aimed at accelerating rare disease diagnosis through genetic newborn screening and AI-based tools. It develops a digital infrastructure for patient engagement, AI algorithms for early detection, and a meta-symptom checker to support faster diagnosis.
**VICT3R** Developing and implementing virtual control groups to reduce animal use in toxicology research https://www.vict3r.eu/	A project aimed at reducing animal use in toxicology research by developing virtual control groups (VCGs) to replace concurrent control groups in non-clinical drug and chemical safety evaluations.

## 2 The Innovative Health Initiative (IHI) collaboration model

The Innovative Health Initiative (IHI)^1^ builds on the Innovative Medicines Initiative (IMI). The IHI expands the IMI’s focus on pharmaceutical research to a broader, cross-sectoral approach to health innovation, addressing evolving scientific and societal needs. It is a unique framework that brings together private health industry sectors (pharma, biotech, vaccines, and medical technologies) and public partners (e.g., academia, healthcare professionals and providers, patients and caregivers, regulators, health technology assessment bodies, and payers) to conduct research in areas of unmet public health need. The IHI, with an overall budget of 2.4 billion EUR, is the largest initiative of its kind in the health space. Funded projects are precompetitive collaborative “consortia,” leveraging at scale the multidisciplinary skills of all members, public European funding, and in-kind industry resources (experts’ time, setting and running of assays and models, clinical studies, etc.) toward the achievement of agreed research objectives. The terms of collaboration and governance in the IHI consortia are established from the start by the Grant Agreement (GA) that all partners must sign with the IHI and by the Consortium Agreement (CA) that all members of the consortium must sign among themselves.^2^ The GA provisions are common for all IHI contracts, while those of the CA are agreed by the consortium members including details for data sharing and access and for intellectual property rights (IPRs). Thus, all partners enter the collaboration with an upfront understanding of their own knowledge/data/assets to be shared with the other collaborators for the implementation of the agreed research program, as well as on rules for access to results and data within and beyond the consortium, guaranteeing respect for the rights of all collaborators and that the collaboration will be rewarding for all involved. Working in the IHI precompetitive trusted framework, collaborating, and sharing data (among companies and with public actors) allows us to learn together to overcome key research and innovation bottlenecks, instead of working in silos. This gives IHI projects the unique ability to access, collate, and make further available an increasing volume and diversity of high-quality data-generating impacts beyond the single projects.

## 3 How the IHI public–private partnerships have enabled unprecedented (big) data access and connectivity

If the large amount of high-quality data existing in the health research ecosystem could be curated, connected, and made accessible for research use, it would unlock unprecedented possibilities for a comprehensive understanding of biological phenomena, patient journeys, treatment paths, and health outcomes. This is not a trivial endeavor due to legal, technical, data protection, ethical, and IPR challenges and requires an enabling legal framework and strong data and knowledge management (DKM).

Under the IHI framework, industries work together in a “safe” precompetitive space, which enables large-scale access to high-quality data generated and collected by industry that is normally siloed within individual companies due to their IPR sensitivity. In addition, DKM has been a central theme from the start in the IMI (3) and even more under the IHI, where one of the overall specific objectives is to “*exploit the full potential of digitalisation and data exchange in healthcare*” (4). In all IHI projects, the data governance and stewardship principles agreed in the consortium agreement are further developed in the *data management plan.* This robust data policy has uniquely enabled data access and connectivity of large amounts of both public and corporate data (including from clinical trials) to create highly curated and standardized databases (e.g., projects DIRECT, EPAD, EUROPAIN, HYPORESOLVE, HIPPOCRATES, NEWMEDS, and projects highlighted later) to address key research questions and deliver high-quality research outputs (5) with impacts in important areas such as Alzheimer’s disease (6) and diabetes (7).

The impact of IHI partnerships goes beyond a single project, creating lasting collaborative networks to sustain and accelerate the progress of data-driven research and deliver new models, solutions, and practices to make the most of the big data potential (8–10).

A pivotal example is from the IHI projects in drug safety. The eTOX project pioneered the “honest broker” approach (11) to integrate historical toxicological data from 13 pharma companies with publicly available data and share them among 30 public and private stakeholders. The approach was so successful that it was extended to the sharing of clinical data in the large-scale follow-up eTRANSAFE (12). eTRANSAFE overall outputs are a reusable framework and provide best practices for sustainable and reproducible findable, accessible, interoperable, and reusable (FAIR) data and knowledge sharing to drive integrative drug safety research at scale. This framework can be adopted by other research communities to integrate public and private data in the biomedical research space, expanding its impact far beyond drug safety (13).

eTRANSAFE also developed the concept of “virtual control groups,” harnessing the wealth of data on control groups in toxicity tests. This will be developed up to regulatory acceptance in the new IHI project VICT3R (14), showing how the IHI framework generates long-lasting collaborative networks to drive sustainable progress in data-driven research.

The MELLODDY project took a different but even more powerful approach to enable the sharing and combining of proprietary datasets without actually sharing them, exposing them, or even moving them from where they are housed, via blockchain and federated learning (FL). MELLODDY created the largest FL-based study across data from 10 pharmaceutical companies and demonstrated the power of combining multiparty privacy-protected machine learning (ML) techniques for cross-end point federated learning in drug discovery, analyzing a dataset of 2.6+ billion confidential experimental activity data points, documenting 21+ million physical small molecules, and 40+ thousand assays in on-target and secondary pharmacodynamics and pharmacokinetics (15). MELLODDY FL model offers a powerful tool to accelerate drug discovery, improve predictive modeling, and potentially reduce time and cost associated with bringing new treatments to market, all while maintaining the highest standards of data security and privacy. The project software outputs and methodologies are available openly via GitHub^3^ for reuse by the research community.

The EMIF project has addressed the challenge of collecting, integrating, and making accessible sensitive patient data: Its platform enabled data access through a federated network of data sources, allowing researchers to find, assess, and reuse health data from a network of 18 population-based and 60 cohort-based data sources across Europe. The platform provided a single point of access for searching aggregated data across different sources and countries. Researchers could browse metadata, assess data suitability, and initiate new research studies with the data sources in a secure environment (16). The EMIF catalog, a key component of the platform, integrated biomedical metadata from multiple institutions using a common metadata schema, allowing researchers to evaluate data source suitability and request access to specific data sources. Data custodians had full control over data visibility levels, ensuring fine-grained access control to the metadata, and a role-based access control system was deployed to enforce EMIF access policies (16). The EMIF project established a sustainable model for sharing healthcare data across Europe, promoting a wider adoption of data-sharing principles (16).

These principles were the basis of the EHDEN project, which was part of the BD4BO program (17). EHDEN built a federated data network for access to the health data of 100 million EU citizens standardized to a common data model. EHDEN has been successful beyond expectations in creating a highly collaborative network of data partners across Europe, supporting them in mapping their data to a common standard and in participating in open science collaborative study-a-thons that have delivered highly significant results (18). These range from demystifying the relation between COVID-19 vaccines and the development of neurological disorders (19) to demonstrating the utility of routinely collected health data to support the validation and prioritization of pharmacovigilance signals (20). Building on EHDEN’s successes, a new legal entity, the EHDEN Foundation,^4^ was created to continue fostering a strong and growing open science community.

A novel area that is spearheaded by the IHI is that of “big” pathology imaging. The BIGPICTURE project is creating the first European ethical and regulatory compliant community-based platform for storing, sharing, and processing millions of quality-controlled annotated pathology images and artificial intelligence algorithms. This required the development of a tailor-made data-sharing agreement to align all 44 public and private partners. The BIGPICTURE platform for digital pathology will drastically accelerate “AI pathology” and accelerate the progress from drug discovery to clinical diagnostics (21).

The learnings on data sharing across the IHI projects are gathered in a Playbook (22) to help future participants in precompetitive consortia in tackling internal processes and decisions and accelerate the provision of, and access to, data from the partners. For example, the Playbook recommends that to navigate the GDPR^5^ consortia should determine data flow and GDPR roles early, consider impact assessments, ensure proper consent, use standardized templates, and address data protection systematically for efficient compliance and sharing. This will create a positive impact on resources, help projects make progress faster, and contribute to a stronger spirit of collaboration.

## 4 How the IHI PPPs are contributing to enhancing data quality and standards

The success of big data-driven research depends critically on the quality of the data. Issues of data quality and standardization could significantly decrease value and lead to significantly biased results. In the IHI partnerships, the robustness of the data generation methodologies and use of standards is a priority to ensure replicability of results [e.g., EUROPAIN (23)], and important resources have been developed to help the broad scientific community to enhance both.

Two examples are the EQIPD project, which delivered a framework for rigor in preclinical animal experiments (24) and training modules available globally via the Partnership for Assessment and Accreditation of Scientific Practice (PAASP),^6^ and the BIGDATA@HEART project, which developed the CODE-EHR, a minimum standards framework to be used by researchers and clinicians to improve the design of studies and enhance the transparency of study methods for robust and effective utilization of healthcare data in research (25).

The FAIR data principles are the basis for the successful sharing and reuse of data in research. The IHI fostered the implementation of these key principles via the FAIRplus project, which has produced a FAIR Cookbook with practical “recipes” for researchers to make their data FAIR (26).

The use of standards and common data models has been key to the success of the IHI projects in making a large amount of data shareable (see previous section) and allowed delivering reliable evidence-based information on important areas such as risks and benefits of medication use in pregnancy (27) and in rare diseases (28). It has also enabled synergies with projects beyond IHI, like in the case of cancer projects, to further boost impact in this critical area (29).

The IHI’s focus on data quality enabled its projects to contribute to the progress of regulatory science (30) and to impactful collaborations with regulatory authorities. A prime instance is again EHDEN, an exemplar open science collaborative consortium whose high level of trustworthiness led to long-term collaboration with the European Medicines Agency (EMA). EHDEN contributed to the infrastructure for real-world monitoring of treatments and vaccines during the COVID-19 pandemic^7^ and now its work is leveraged for the establishment of the Coordination Centre for the Data Analysis and Real-World Interrogation Network (DARWIN EU®).^8^

## 5 IHI PPPs boost progress in big data research in areas of high public health need: the example of neurodegeneration, autism, and cancer

A research area where globally there has been massive investment in both public funding and industry resources is that of neurodegeneration. This has generated a huge body of data, both preclinical and patient-derived. However, such data resources are highly fragmented, difficult to access, poorly standardized, and of variable quality. Federated data-sharing approaches can address at least some of these issues, as shown by the IHI and others (31, 32). However, a more fit-for-purpose and collaborative framework is needed to enable access across public and private data collections to significantly boost the availability of data for federated analysis. The NEURONET project gathered important learnings across the IHI portfolio of neurodegenerative projects, identifying as enablers the principles of transparency, standardization, and co-design—from open, accessible metadata catalogs that enhance findability of data, to measures that increase visibility and trust in data reuse (10). The EPND project (33) is going a step further in developing a scalable and sustainable platform for high-quality sample and data sharing in neurodegeneration. Importantly, EPND leverages the existing data platform ADDI^9^ AD Workbench to connect to an existing global network of data scientists and datasets. This highly collaborative approach further promotes open innovation and the generation of additional resources for the research community and, in doing so, speeds up research progress in neurodegeneration.

Despite the potential promise of big data in psychiatry, the sharing of data in this area has long posed ethical and logistical challenges due to research data capturing human characteristics and experiences, often including sensitive information, such as demographic factors and genetic data. These issues are further amplified in child and adolescent psychiatry, where key decisions regarding research participation and data use may be made by a legal guardian on behalf of a young person. The EU-AIMS and follow-up AIMS-2-TRIALS projects have succeeded in tackling these challenges using a powerful combination of striving for data quality and using mechanisms for shared control of data curation between researchers and participants such as dynamic consent (34). They created the largest collection of multimodal longitudinal data in autism (35), the EAGER registry (36), including 1,500 re-contactable participants with a diagnosis of autism or associated rare genetic condition who have undergone whole-genome sequencing, and aim to make both data networks globally shareable.^10^

Cancer is another key area where big data applications can help improve patients’ quality of life if patient data can be gathered with sufficient high quality and respect for regulations. To this end, the IHI has created several best-practice data platforms for different malignancies. The HARMONY Alliance (blood cancer) and PIONEER (prostate cancer) platforms include large amounts of harmonized and anonymized data, plus data processing pipelines and analytics ensuring data safety and quality, as well as patient privacy, allowing scientists to answer research questions that cannot be addressed with other methods (37–40). The OPTIMA project (prostate, breast, and lung cancer) is establishing a secure, interoperable, large-scale evidence, GDPR-compliant data platform that includes RWD from >200 million people, data analysis tools, FL tools, and AI algorithms to identify factors supporting individualized care decisions for oncologic patients (41). The recent IDERHA (lung cancer) infrastructure connects existing data providers’ resources (low and high-dimensional data) so users can access and analyze the data compliant with data standards, GDPR, and regulatory requirements, enabling secondary use of data while ensuring full control by citizens of their personal health data. IDERHA is building one of the first pan-European health data spaces, aligned with the implementation of the upcoming European Health Data Space (EHDS) (42).

## 6 Discussion

The IHI collaboration model has demonstrated significant success in enabling large-scale data sharing and connectivity in health research. By creating a trusted precompetitive framework, the IHI has overcome key bottlenecks in this challenging domain and has generated a significant body of high-quality (big) data, as well as infrastructures and learnings for fostering progress in various health areas and enabling faster, more robust innovation while respecting the rights of all stakeholders, particularly patients. Having the patients as active partners in all data activities and decisions on data sharing/access has been essential to justify their transparency and legitimacy (39, 43, 44), ensuring research answers questions that really match the need of the affected people, ultimately boosting the quality of its outputs. This approach is giving fruit as high-quality results and impacts, including showing how big data analytics can dramatically accelerate and make more robust evidence generation to inform clinical decision-making for better patient health outcomes (45, 46). The high scientific quality of the teams, public–private cross-learning, and careful design and monitoring of the activities ensure the project objectives keep and often go beyond the innovation state-of-the-art.

Many other funders and projects are following this path, but the IHI has done it at scale, and the results of the projects are fostering sustainable ecosystems to carry on learning and innovating, as flagged in the most recent evaluation report by the European Commission (47).

IHI partnerships prioritize data quality, standards, and the use of common data models for interoperability. These practices enable the delivery of trustworthy results and a robust evidence base for translating research into improved patient care practices and products, including contributions to progress in regulatory science.

The IHI journey in the big data area is only at the beginning, and many challenges remain. For example, federated learning (FL) has shown promising results in projects such as EHDEN and MELLODDY, leveraging privacy-preserving technologies and adhering to standards, regulations, and ethical requirements. However, significant work remains to be done to fully realize FL’s potential and expand its adoption. A focus is on optimizing the use of real-world data (RWD), including RWD generated by medical devices. A recent multistakeholder workshop at the IHI stressed that growing regulatory science, building on transparency, trust, and legitimacy, improving standardization, boosting data and digital literacy, and building on already existing infrastructures are key to boosting progress in RWD use in healthcare.^11^ These are ideal areas for future IHI partnerships.

## Data Availability

Publicly available datasets were analyzed in this study. This data can be found here: this perspective describe multiple projects results from IHI projects details can be found via https://www.ihi.europa.eu/projects-results.

## Footnotes

^1^Innovative Health Initiative (IHI). https://www.ihi.europa.eu/; for simplicity, we will use IHI also to cover IMI.

^2^ https://www.ihi.europa.eu/apply-funding/call-documents

^3^ https://github.com/melloddy

^4^ https://www.ehden.eu/vision-and-mission/ehden-legal-entity/

^5^GDPR: General Data Protection Regulation.

^6^ https://paasp.net/

^7^ https://www.ema.europa.eu/en/news/covid-19-ema-sets-infrastructure-real-world-monitoring-treatments-and-vaccines

^8^ https://www.ehden.eu/darwin-eu-emc/

^9^Alzheimer’s Disease Data Initiative ADDI https://www.alzheimersdata.org/

^10^ https://www.aims-2-trials.eu/our-research/data-management-analysis/

^11^Report of the workshop will be available in 2025 on the IHI website.
